# Dysregulation of Respiratory Center Drive (P0.1) and Muscle Strength in Patients With Early Stage Idiopathic Parkinson's Disease

**DOI:** 10.3389/fneur.2019.00724

**Published:** 2019-07-03

**Authors:** Wei Zhang, Lei Zhang, Ning Zhou, Enqiang Huang, Qi Li, Tongyu Wang, Chunchao Ma, Bin Li, Chen Li, Yanfen Du, Jing Zhang, Xiaofeng Lei, Alysia Ross, Hongyu Sun, Xiaodong Zhu

**Affiliations:** ^1^Department of Neurology, Tianjin Neurological Institute, Tianjin Medical University General Hospital, Tianjin, China; ^2^Department of Respiratory, Tianjin Medical University General Hospital, Tianjin, China; ^3^Neurology Department of PKU Care CNOOC Hospital, Beijing, China; ^4^Department of Neurology, Tianjin First Central Hospital, Tianjin, China; ^5^Department of Neurology, Tianjin Haibin People's Hospital, Tianjin, China; ^6^Department of Neurology, The Fifth Central Hospital of Tianjin, Binhai Hospital of Peking University, Tianjin, China; ^7^Department of Neurology, The Second Hospital of Tianjin Medical University, Tianjin, China; ^8^Department of Neurology, Tianjin Third Central Hospital, Tianjin, China; ^9^Department of Neurology, Tianjin Fourth Center Hospital, Tianjin, China; ^10^Department of Neuroscience, Carleton University, Ottawa, ON, Canada

**Keywords:** Parkinson's disease, respiratory center drive, respiratory muscle strength, P0.1, biomarker

## Abstract

**Objective:** The goal of this study is to evaluate pulmonary function and respiratory center drive in patients with early-stage idiopathic Parkinson's disease (IPD) to facilitate early diagnosis of Parkinson's Disease (PD).

**Methods:** 43 IPD patients (Hoehn and Yahr scale of 1) and 41 matched healthy individuals (e.g., age, sex, height, weight, BMI) were enrolled in this study. Motor status was evaluated using the Movement Disorders Society-Unified PD Rating Scale (MDS-UPDRS). Pulmonary function and respiratory center drive were measured using pulmonary function tests (PFT). All IPD patients were also subjected to a series of neuropsychological tests, including Non-Motor Symptoms Questionnaire (NMSQ), REM Sleep Behavior Disorder Screening Questionnaire (RBDSQ), Beck Depression Inventory (BDI) and Mini Mental State Examination (MMSE).

**Results:** IPD patients and healthy individuals have similar forced vital capacity (FVC), forced expiratory volume in 1s (FEV1), forced expiratory volume in 1s/forced vital capacity (FEV1/FVC), peak expiratory flow (PEF), and carbon monoxide diffusion capacity (DLCOcSB). Reduced respiratory muscle strength, maximal inspiratory pressure (PImax) and maximal expiratory pressure (PEmax) was seen in IPD patients (*p* = 0.000 and *p* = 0.002, respectively). Importantly, the airway occlusion pressure after 0.1 s (P0.1) and respiratory center output were notably higher in IPD patients (*p* = 0.000) with a remarkable separation of measured values compared to healthy controls.

**Conclusion:** Our findings suggest that abnormal pulmonary function is present in early stage IPD patients as evidenced by significant changes in PImax, PEmax, and P0.1. Most importantly, P0.1 may have the potential to assist with the identification of IPD in the early stage.

## Introduction

Parkinson's disease (PD) is the second most common chronic and progressive neurodegenerative disease in the elderly (1). Although the pathogenesis of PD has been redefined with triggers (e.g., viral infections or environmental toxins), facilitators (e.g., systemic inflammation, mitochondrial dysfunction, and genetic facilitators), and aggravators (e.g., impaired autophagy, neuroinflammation), there is a complex interplay of genetic, aging, and environmental factors that result in PD (2). Consequently, clinical challenges remain because it is difficult to provide a definitive diagnosis at the earliest stage. This has led to an urgent yet unmet need to find a useful examination to facilitate the early diagnosis of PD (3).

Pulmonary function impairment has been studied in idiopathic Parkinson's disease (IPD), the most common type of Parkinsonism whereby the cause is unknown (4). Pulmonary dysfunction has been known as an autonomic disorder equated to non-motor symptoms of PD in several studies. Abnormalities involving obstructive, restrictive and mixed-type pulmonary dysfunction increases disability in PD patients (5). Moreover, respiratory complications, such as aspiration pneumonia and pulmonary embolism are associated with mortality of PD patients (6). A systematic review and meta-analysis suggested asymptomatic respiratory impairment can be discovered even in the initial stages of the disease course (7). In the past decades, research on PD and lung function has brought some meaningful discoveries. Based on the experimental designs and conclusions, these studies can be classified into four main topics: first is the effect of Levodopa (combined with different formulation) on pulmonary dysfunction in PD; the second is about dysregulated lung function, especially ventilatory dysfunction, diffusion impairment, obstructive, and restrictive disorders in PD; the third is the potential relationship between swallowing impairment and respiratory dysfunction in PD; the last is the therapeutic effect of rehabilitation (e.g., YOGA, Qigong) on pulmonary function in patients with PD (8–12). It is worth mentioning that most of these studies were performed in patients in the advanced stage of PD. Currently, whether pulmonary function is affected in early stage IPD patients remains elusive.

As a valid measurement of respiratory center drive, the airway occlusion pressure (P0.1) is a simple, convenient, non-invasive measurement index. It is described as the pressure developed in the occluded airway 100 ms after the onset of inspiration (13). P0.1 is commonly monitored in respiratory diseases, along with respiratory failure and chronic obstructive pulmonary disease (COPD). It is a sensitive indicator of respiratory drive to predict extubation failure in ICU (14). However, respiratory center drive in early IPD has never been studied.

In the present study, we therefore evaluated respiratory center drive and pulmonary function in early-stage IPD patients. Specifically, we examined the pulmonary ventilation function, respiratory muscle strength index, and respiratory drive in early IPD patients and healthy controls.

## Subjects/Materials and Methods

### Human Subjects

In this study, a total of 43 IPD patients (19 male, 24 female; mean age 62.60 ± 6.60 years) and 41 healthy control subjects (21 male, 20 female; mean age 61.39 ± 5.87 years) were enrolled from May 2017 to September 2018 at the Department of Neurology at the General Hospital, Tianjin Medical University, Tianjin, China. The main clinical characteristics of the study population are summarized in Table 1. There were no significant differences in the age, height, weight, and BMI between IPD patients and healthy controls (Table 2). All IPD patients have a mean disease duration of 1.67 ± 1.14 years and received regular dopaminergic treatment with a mean total levodopa equivalent dose (LED) 313.18 ± 206.50 mg according to conversion factors among dopamine drugs recommended by the Movement Disorder Society (15). All subjects signed written informed consent before being recruited for the study. The study was approved and performed in accordance with the guidelines of the Ethics Committee of the General Hospital of Tianjin Medical University.

**Table 1:** Overview of the demographic and clinical characteristics of PD patients and healthy controls.

**PD patients**						**Healthy controls**	
**Case#**	**Age (years, range)**	**Diagnosis**	**Disease duration (years)**	**Hoehn and Yahr scale**	**Levodopa equivalent doses (LED, mg)**	**Case#**	**Age (years, range)**
1	50–60	IPD	1	1	50.00	1	60–70
2	60–70	IPD	2	1	575.00	2	60–70
3	60–70	IPD	1	1	375.00	3	70–80
4	50–60	IPD	2	1	100.00	4	60–70
5	50–60	IPD	2	1	531.95	5	60–70
6	60–70	IPD	1	1	190.98	6	70–80
7	60–70	IPD	0.5	1	525.00	7	60–70
8	60–70	IPD	4	1	350.00	8	60–70
9	60–70	IPD	2	1	525.00	9	60–70
10	50–60	IPD	2	1	50.00	10	60–70
11	60–70	IPD	3	1	237.50	11	60–70
12	60–70	IPD	1	1	437.50	12	60–70
13	60–70	IPD	2	1	250.00	13	60–70
14	70–80	IPD	1	1	375.00	14	60–70
15	50–60	IPD	1	1	50.00	15	60–70
16	50–60	IPD	1	1	250.00	16	60–70
17	50–60	IPD	2	1	237.50	17	60–70
18	60–70	IPD	1	1	187.50	18	60–70
19	70–80	IPD	1	1	400.00	19	50–60
20	70–80	IPD	1	1	412.50	20	60–70
21	50–60	IPD	0.5	1	87.50	21	60–70
22	60–70	IPD	1	1	50.00	22	50–60
23	50–60	IPD	2	1	287.50	23	60–70
24	60–70	IPD	3	1	575.00	24	50–60
25	60–70	IPD	1	1	350.00	25	50–60
26	60–70	IPD	0.5	1	375.00	26	50–60
27	60–70	IPD	3	1	312.50	27	50–60
28	50–60	IPD	1	1	50.00	28	60–70
29	40–50	IPD	2	1	75.00	29	40–50
30	60–70	IPD	2	1	768.75	30	50–60
31	60–70	IPD	0.5	1	50.00	31	50–60
32	60–70	IPD	4	1	550.00	32	50–60
33	60–70	IPD	5	1	725.00	33	60–70
34	50–60	IPD	2	1	375.00	34	50–60
35	60–70	IPD	1	1	750.00	35	60–70
36	60–70	IPD	1	1	412.50	36	50–60
37	70–80	IPD	5	1	525.00	37	50–60
38	50–60	IPD	1	1	287.50	38	60–70
39	60–70	IPD	1	1	50.00	39	50–60
40	70–80	IPD	1	1	87.50	40	60–70
41	60–70	IPD	1	1	375.00	41	50–60
42	60–70	IPD	1	1	187.50		
43	60–70	IPD	1	1	50.00		

*As illustrated in Table 1, 43 idiopathic Parkinson's disease (IPD) patients (19 male and 24 female) with Hoehn and Yahr scale of 1 and 41 healthy controls (21 male and 20 female) enrolled in this study*.

**Table 2:** Demographic and clinical data of PD patients and healthy controls and NPF group.

	**PD (*N* = 43)**	**Healthy controls (*N* = 41)**	***p***
Gender (male/female)	19/24	21/20	0.519
Age (years)	62.60 ± 6.60	61.39 ± 5.87	0.376
Height (m)	1.62 ± 0.07	1.65 ± 0.08	0.060
Weight (kg)	64.81 ± 10.72	66.15 ± 11.85	0.591
BMI	24.67 ± 3.32	24.23 ± 3.32	0.567
Disease duration (years)	1.67 ± 1.14	NA	NA
MDS-UPDRS	15.58 ± 5.95	NA	NA
NMSQ	7.37 ± 3.89	NA	NA
RBDSQ	2.88 ± 3.16	NA	NA
BDI	7.16 ± 8.08	NA	NA
MMSE	28.11 ± 1.82	NA	NA

*As illustrated in Table 2, the PD group and healthy control (HC) group had similar mean ages, gender, height, weight, and Body Mass Index (BMI) (all p > 0.05). MDS-UPDRS, Movement Disorder Society—Unified Parkinson's Disease Rating Scale; NMSQ, non-motor symptoms questionnaire; RBDSQ, REM Sleep Behavior Disorder Screening Questionnaire; BDI, Beck Depression Inventory; MMSE, Mini-Mental State Examination*.

#### Inclusion Criteria of the PD Patients

All patients were diagnosed with IPD based on the clinical criteria of the United Kingdom PD Society Brain Bank (16). Motor status was evaluated using the Movement Disorders Society-Unified Parkinson's Disease Rating Scale (MDS-UPDRS). The Hoehn and Yahr stages of all patients were 1 (H&Y stage = 1), clinical signs and symptoms were unilateral involved only, usually with minimal or no functional impairment (17), and all these PD patients had no fluctuations in symptoms, such as on-off phenomena.

#### Exclusion Criteria of the PD Patients

IPD patients were excluded under the following conditions: (1) Suffering from any clinical neurological, respiratory, cardiovascular, or other systemic diseases verified by specialized physicians through clinical and appropriate instrumental diagnosis, such as cerebral infarction, cerebral hemorrhage, encephalitis, Myasthenia Gravis, COPD, asthma, emphysema, respiratory tract infection, anemia, coronary heart disease etc.; (2) Complaining of any heart and lung discomfort (e.g., dyspnea, palpitation, etc.); (3) History of smoking or lung, brain or cardiac surgery trauma; (4) Receiving any treatments (e.g., β-blocker, sedatives, hypnotics, antibiotic, NAIDS, etc.) that may influence pulmonary function; (5) Severe cognitive dysfunction or dementia according to the Mini-Mental State Examination (MMSE < 25) (18); (6) Other causes leading to the inability to tolerate the study or factors affecting lung function.

#### Requirements for Healthy Subjects

The healthy controls were age, sex, and BMI matched with all PD patients. They were in healthy condition at the time of the study and were screened for the same exclusion criteria as the PD patients.

### Instruments and Procedures

All the patients and healthy controls underwent pulmonary function tests (PFT) according to the standards set by the American Thoracic Society (ATS) under the guidance of a respiratory physician. The apparatus used in this study included a spirometer and a respiratory actuator (MasterScreen, JAEGER, Germany). To ensure research consistency, fixed check time (8:00–12:00 a.m.), place (in a quiet environment, reducing the impact on subjects), and indoor temperature (24–28°C) were kept constant. Before the test, these early-stage PD patients were required to stop the associated anti-Parkinson's disease treatment at least 12 h to avoid the impact of drugs on testing. Beyond that, all patients underwent related neuropsychological assessments. Parameters related to PFT and various symptom scales are as follows:

#### Parameters Related to PFT

Predicted values were automatically generated by the PFT equipment according to the height, weight and age of the examinees before the test. Following examination, the percentages of the measured value from the test compared to the predicted value from the computer were automatically calculated. In this study, these percentages were considered as the PFT measurements to be recorded and processed further.

#### Ventilation Function Indicators

The percentages of the predicted values of forced vital capacity (FVC%), forced expiratory volume in 1s (FEV1%), forced expiratory volume in 1s/forced vital capacity (FEV1/FVC%), peak expiratory flow (PEF%), total lung capacity (TLC%), residual volume (RV%), carbon monoxide diffusion capacity (DLCOcSB%) were evaluated as indicators of ventilation function.

#### Respiratory Muscle Strength Index

The percentages of the predicted values of maximal inspiratory pressure (PImax%) and maximal expiratory pressure (PEmax%) were measured to determine the respiratory muscle strength.

#### Respiratory Drive Index

The percentage of the predicted value of the airway occlusion pressure (P0.1%) was examined to determine the respiratory drive.

### Neuropsychological Examination

To assess neuropsychological status, all patients also underwent the Non-Motor Symptoms Questionnaire (NMSQ), REM sleep behavior Disorder Screening Questionnaire (RBDSQ), Beck Depression Inventory (BDI), and Mini Mental State Examination (MMSE).

### Statistical Analysis

Statistical analyses were performed using SPSS 22.0 (IBM, USA). All the summary results were presented as the mean ± standard deviation (SD). All data was first checked for any potential outliers and tested for normality using the Shapiro-Wilk normality test. Data Significance was then assessed by student's unpaired *t*-test for data with normal distributions. A value of *p* < 0.05 was considered to be statistically significant.

## Results

### Unaltered Pulmonary Ventilation Function in Early-Stage IPD Patients

**Figure 1:**
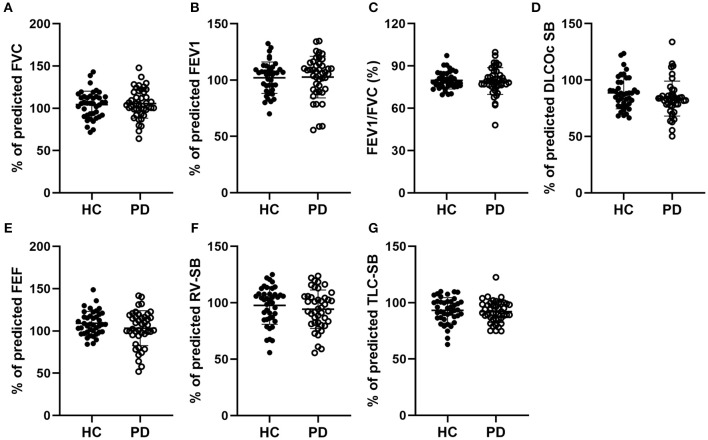
Comparisons of the pulmonary ventilation function between early-stage PD patients and age-matched healthy controls. **(A–G)** Percentage of predicted values of FVC **(A)**, FEV1 **(B)**, FEV1/FVC **(C)**, DLCOc SB **(D)**, FEF **(E)**, RV-SB **(F)**, and TLC-SB **(G)** showed no significant difference between early-stage PD patients (open circles, *n* = 43) and age-matched healthy controls (filled circles, *n* = 41, *p* > 0.05). Each symbol represents a single subject. Data are presented as mean ± SD.

We first assessed the pulmonary ventilation function in early-stage IPD patients and healthy controls by measuring FVC (%), FEV1 (%), FVC/FEVF1 (%), PEF (%), RV (%), TLC (%), and DLCOcSB (%) (Figure 1). Consistent with the early clinical stage of these IPD patients, we found no significant changes in ventilation function in IPD patients (FVC: 105.51 ± 17.24%; FEV1: 102.67 ± 18.50%; FVC/FEVF1: 79.27 ± 9.46%; PEF: 103.19 ± 20.67%; RV: 94.26 ± 17.09%; TLC: 92.04 ± 9.67%; DLCOcSB: 83.57 ± 15.55%; *n* = 43) compared to healthy controls (FVC: 104.38 ± 15.90%, *p* = 0.76; FEV1: 102.12 ± 13.93%, *p* = 0.88; FVC/FEVF1: 79.71 ± 6.10%, *p* = 0.80; PEF: 109.01 ± 13.99%, *p* = 0.13; RV: 97.64 ± 16.65%, *p* = 0.36; TLC: 93.21 ± 11.26 %, *p* = 0.61; DLCOcSB: 88.63 ± 14.09%, *p* = 0.12; *n* = 41). In addition, there were no significant correlations between pulmonary ventilation function tests and neuropsychological assessments (*p* > 0.05, Table 3).

**Table 3:** Correlation analysis of pulmonary function tests (PFT) values and neuropsychological assessments.

		**MDS-UPDRS**	**NMSQ**	**RBDSQ**	**BDI**	**MMSE**
FVC (%)	*r*	−0.04	0.13	−0.04	0.02	−0.08
	*P*	0.81	0.40	0.83	0.15	0.61
FEV1 (%)	*r*	−0.20	0.17	0.05	0.12	0.07
	*P*	0.20	0.27	0.74	0.44	0.68
FVC/FEV1(%)	*r*	−0.27	0.10	0.15	−0.23	0.07
	*P*	0.08	0.51	0.33	0.15	0.68
PEF (%)	*r*	−0.23	−0.00	0.00	0.03	0.35
	*P*	0.13	0.99	0.99	0.86	0.06
RV (%)	*r*	−0.22	−0.24	−0.18	−0.19	−0.04
	*P*	0.15	0.12	0.24	0.21	0.79
TLC (%)	*r*	−0.25	−0.09	−0.04	0.13	−0.07
	*P*	0.11	0.59	0.82	0.41	0.66
DLCOcSB(%)	*r*	−0.16	−0.01	−0.11	−0.21	0.08
	*P*	0.32	0.93	0.49	0.18	0.63
PImax (%)	*r*	−0.02	−0.01	0.06	0.00	0.17
	*P*	0.88	0.97	0.70	0.99	0.27
PEmax (%)	*r*	−0.12	0.13	0.10	−0.12	0.03
	*P*	0.43	0.42	0.52	0.45	0.84
P0.1 (%)	*r*	0.12	−0.08	−0.10	−0.15	0.88
	*P*	0.45	0.63	0.53	0.33	0.59

*As illustrated in Table 3, there was no significant correlation between pulmonary function tests values and neuropsychological assessments*.

### Decreased Respiratory Muscle Strength in Early-Stage IPD Patients

**Figure 2:**
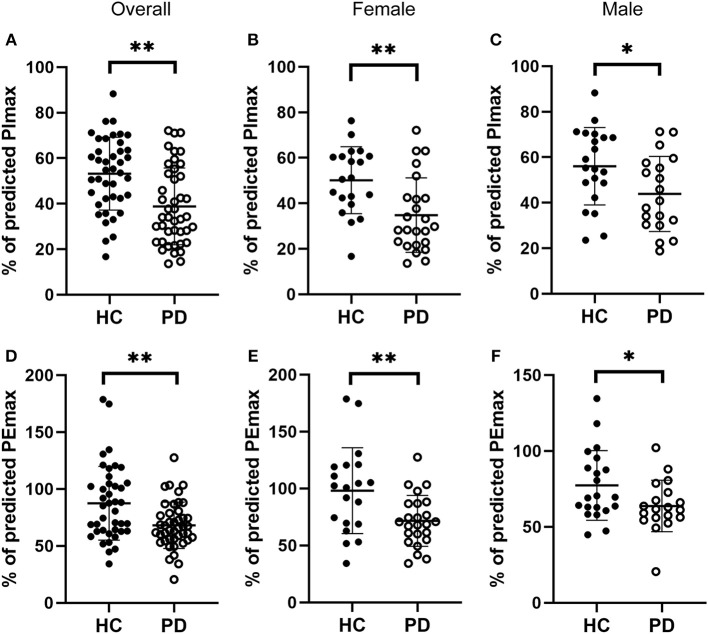
Comparisons of the respiratory muscle strength between early-stage PD patients and age-matched healthy controls. **(A–C)** Percentage of predicted values of maximal inspiratory pressure [PImax, **(A)**] was significantly lower in early-stage PD patients (open circles, *n* = 43) and age-matched healthy controls (filled circles, *n* = 41, **p < 0.001). Note that both female [**(B)**, *n* = 24] and male [**(C)**, *n* = 19] early-stage PD patients (open circles) showed significantly lower values of PImax compared to the corresponding healthy controls (filled circles; female *n* = 20, male *n* = 21, *p < 0.05, **p < 0.01). **(D–F)** Percentage of predicted values of maximal expiratory pressure [PEmax, **(D)**] was significantly lower in early-stage PD patients (open circles, *n* = 43) and age-matched healthy controls (filled circles, *n* = 41, **p < 0.001). Note that both female [**(E)**, *n* = 24) and male [**(F)**, *n* = 19] early-stage PD patients (open circles) showed significantly lower values of PEmax compared to the corresponding healthy controls (filled circles; female *n* = 20, male *n* = 21, *p < 0.05, **p < 0.01). Each symbol represents a single subject. Data are presented as mean ± SD.

We next evaluated respiratory muscle strength by assessing muscle strength index PImax and PEmax in both early-stage IPD patients and healthy controls. As shown Figures 2A,D, the early-stage IPD patients showed a significant reduction in both PImax (38.82 ± 16.87%, *n* = 43) and PEmax (68.13 ± 20.31%, *n* = 43) compared to healthy controls (PImax: 53.17 ± 16.00%, *n* = 41, *p* = 0.001; PEmax: 87.49 ± 32.46%, *n* = 41, *p* = 0.002). No significant correlations were found between PImax and neuropsychological assessments (MDS-UPDRS: *p* = 0.08, *r* = −0.02; NMSQ: *p* = 0.97, *r* = −0.01; RBDSQ: *p* = 0.70, *r* = 0.06; BDI: *p* = 0.99, *r* = 0.00; MMSE: *p* = 0.27, *r* = 0.17; Table 3), or between PEmax and neuropsychological assessments (MDS-UPDRS: *p* = 0.43, *r* = −0.12; NMSQ: *p* = 0.42, *r* = 0.13; RBDSQ: *p* = 0.52, *r* = 0.10; BDI: *p* = 0.45, *r* = −0.12; MMSE: *p* = 0.84, *r* = 0.03; Table 3). To further determine whether there is any sex-specific difference, we performed subgroup analysis by sex (Figures 2B,C,E,F). We found that both female and male early-stage IPD patients show a significant decrease in PImax (Female PD patients: 34.82 ± 16.39%, *n* = 24; Male PD patients: 43.87 ± 16.52%, *n* = 19, Figures 2B,C) and PEmax (Female PD patients: 71.60 ± 22.39%, *n* = 24; Male PD patients: 63.73 ± 16.91%, *n* = 19, Figures 2E,F) compared to female (PImax: 50.15 ± 14.73%, *n* = 20, *p* = 0.0024; PEmax: 98.16 ± 37.77%, *n* = 20, *p* = 0.006) and male (PImax: 56.06 ± 16.98%, *n* = 21, *p* = 0.027; PEmax: 77.33 ± 23.02%, *n* = 21, *p* = 0.042) healthy controls.

### Increased Respiratory Drive in Early-Stage IPD Patients

**Figure 3:**
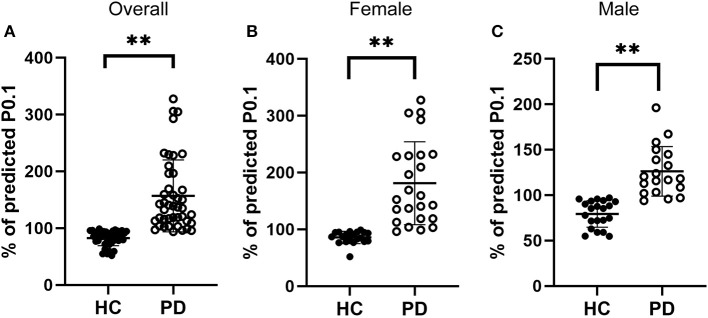
Comparisons of the respiratory drive between early-stage PD patients and age-matched healthy controls. **(A)** Percentage of predicted values of respiratory center drive (P0.1), was significantly higher in early-stage PD patients (open circles, *n* = 43) and age-matched healthy controls (filled circles, *n* = 41, **p < 0.001). **(B–C)** Both female [**(B)**, *n* = 24] and male [**(C)**, *n* = 19] early-stage PD patients (open circles) showed significantly larger values of PImax compared to the corresponding female [**(B)**, *n* = 20] and male [**(C)**, *n* = 21] healthy controls (filled circles, **p < 0.01). Each symbol represents a single subject. Data are presented as mean ± SD.

Respiratory center drive, P0.1, is associated with the level of muscular inspiratory activity. As we found significant decreases in respiratory muscle strength, we next aimed to assess respiratory center drive in early-stage IPD patients as well as healthy controls (Figure 3). Indeed, we found that early-stage IPD patients showed significant increases in respiratory center drive P0.1 (156.78 ± 63.24%, *n* = 43) compared to age-matched controls (82.40 ± 13.14%, *n* = 41, *p* < 0.001, Figure 3A). Importantly, both female and male early-stage IPD patients show a significant increase in P0.1 (Female PD patients: 181.1 ± 73.00%, *n* = 24; Male PD patients: 126.1 ± 27.31%, *n* = 19, Figures 3B,C) compared to female (85.70 ± 10.78%, *n* = 20, *p* = 0.001) and male (79.26 ± 14.61%, *n* = 21, *p* = 0.001) healthy controls. It is worth mentioning that respiratory center drive P0.1 values of most male and female IPD patients were indeed separated from control values with minimal overlap. No significant correlations were found between P0.1 and neuropsychological assessments (MDS-UPDRS: *p* = 0.45, *r* = 0.12; NMSQ: *p* = 0.63, *r* = −0.08; RBDSQ: *p* = 0.53, *r* = −0.10; BDI: *p* = 0.33, *r* = −0.15; MMSE: *p* = 0.59, *r* = 0.88; Table 3).

## Discussion

The present study provides the first evidence of abnormal pulmonary function in the very early stage of IPD. As documented here, patients with early IPD had significantly lower respiratory muscle strength (PImax%, PEmax%) and higher central respiratory drive (P0.1%) than those in age-matched healthy controls. Importantly, the values of measured respiratory center drive P0.1 showed a remarkable separation between the early-stage IPD patients and healthy controls, indicating its potential utility for diagnosis of the early-stage IPD patients.

Abnormalities of peak expiratory and inspiratory flows (PEF, PIF) were found in patients with relatively severe PD and without clinical signs or symptoms of respiratory problem. These changes can be explained by “muscle weakness” and hypokinesia, two symptoms intrinsic to PD (19). What's more, levodopa improved significant variations in PEF by decreasing tremor and muscle rigidity, increasing coordination of muscles, and facilitating movement (20). In previous studies, upper airway dysfunction and a restrictive pattern are the main spirometric findings in PD. The main reasons might be a poor coordination or rigidity of respiratory muscles, which could limit forced respiratory movements (21). Unlike many previous studies, our data did not show any respiratory abnormalities including ventilation or diffusion dysfunction. This may be because the subjects in our study were very early stage patients (H&Y scale of 1) rather than more severe patients (H&Y scale of 2 or higher). Respiratory muscle strength, assessed by measuring the maximal inspiratory and expiratory mouth pressures (PImax and PEmax) was decreased in IPD patients. Furthermore, all these parameters of respiratory muscle performance tended to increase in the “on” period rather than the “off” period, although this increase did not reach statistical significance (22). This was due to poor posture restricting chest and abdominal movements, rigidity resulting in poor chest wall compliance and disrupted respiratory muscle coordination (23). Consistent with previous studies, our findings that the values of PImax and PEmax in early-stage IPD were significantly lower than the healthy controls suggest that inspiratory muscle strength appears to be impaired in IPD patients at very early stage (24). As previously thought, PD involves dysfunction in both movement and muscle tone in the trunk and limbs related to abnormal thoracoabdominal movements as well as incoordination of respiratory muscle (25).

Respiratory center drive, or P0.1, is associated with the level of muscular inspiratory activity. It involves the nerve impulse that the respiratory center gives out to stimulate the inspiratory muscle to contract when we inhale. It is mainly affected by sensitivity of chemoreceptors *in vivo*, partial pressure of arterial oxygen CO2, resistance to inhalation, and abnormal function of inspiratory muscles (26). The output of the respiratory center can be estimated by many parameters, such as ventilation, inspiratory muscle power or EMG of the diaphragm. However, P0.1, the airway occlusion pressure (the negative airway pressure generated during the first 100 ms of an occluded inspiration) represents the most important index of the output of the respiratory centers (27). In many studies, P0.1 is a useful parameter in setting the level of pressure support ventilation in mechanical ventilation (28). Additionally, P0.1 can be used as a good indicator of postextubation respiratory distress after extubation (29). The most striking finding of our study was significant changes in respiratory center drive in patients with early stage IPD. The percentage of the predicted values of P0.1 (P0.1%) in IPD group was significantly higher than in the control group (*p* < 0.001). In our study, factors such as respiratory diseases or smoking which can influence P0.1 were excluded, suggesting that the abnormal respiratory drive was, therefore, mainly related to IPD itself. Based on relevant literature, there are two possible mechanisms at play. First is abnormal respiratory muscle strength of PD patients (peripheral mechanisms). Second is abnormalities in the respiratory center itself (central mechanisms). Abnormalities of the chest wall or its musculature can change lung volumes without necessarily altering expiratory flow (30). PD with a restrictive pattern of pulmonary dysfunction could probably be explained by abnormally low chest wall compliance secondary to chest wall rigidity (31). As described in the diagnostic criteria, as PD patients manifest their core symptoms, such as rigidity and bradykinesia, their movements become slow, clumsy, inflexible and uncoordinated, which impairs the tone, contractility, and coordination of thoracic musculature. As a result, the respiratory mechanics and pulmonary function are affected. The decrease of respiratory muscle force may cause an increase in compensation of central respiratory driving force to maintain adequate physiological ventilation. This central self-regulation mechanism is common in respiratory diseases such as COPD and asthma (32). In our study, the H&Y scale of IPD patients is 1 (the course of illness is extremely short), so the impairment of common lung ventilation (restrictive, obstructive, and mixed), is not evident or within the scope of compensation. So, P0.1 is a useful and sensitive measurement in early-stage IPD (28). Another mechanism involved is the problem with the respiratory center itself. Based on the Braak hypothesis, which suggests that alpha-synuclein aggregation starts from olfactory bulb and dorsal vagus nucleus, and develops gradually up and forward along the brain stem, there is early brainstem involvement in PD patients (33). Some researchers suggest that there is a depletion of the chemosensitive glutamatergic neurons in the dorsolateral pons and the ventrolateral medulla in both PD and MSA patients, which may trigger the impairment of respiratory muscle control (34). In a recent study by Lee et al. involvement of the central autonomic network and gray matter loss may underlie the respiratory dysfunction in PD patients (35). In addition, autonomic nerve dysfunction, such as vagus nerve damage, leads to increased upper airway muscle tone, resulting in uncoordinated local movements and weak muscular contraction (36, 37). There is currently little evidence of brainstem-mediated respiratory dysfunction, however it seems reasonable that as PD progresses along with further loss of dopaminergic cells in the midbrain, basal ganglia and medulla, respiratory function and ventilation will deteriorate (38).

The early diagnosis of PD will greatly help optimize therapeutic strategies, especially in cases with very mild or a-typical motor and non-motor symptoms. That being said, we have found significant increases in respiratory center drive (P0.1) and decreases in respiratory muscle strength PImax and PEmax in early-stage IPD patients. Importantly, measured P0.1 values in both female and male early-stage IPD patients were remarkably separated from those in healthy controls, while measured PImax and PEmax values showed moderate overlap between early-stage IPD patients and healthy controls. Therefore, our data strongly supports P0.1 as a safe, non-invasive and convenient measurement for early-stage IPD in clinical practice.

## Data Availability

All datasets generated for this study are included in the manuscript and/or the supplementary files.

## Ethics Statement

This study was carried out in accordance with the recommendations of the Ethics Committee of the General Hospital of Tianjin Medical University with written informed consent from all subjects. All subjects gave written informed consent in accordance with the Declaration of Helsinki. The protocol was approved by the Ethics Committee of the General Hospital of Tianjin Medical University.

## Author Contributions

WZ, LZ, HS, and XZ: designed experiments. WZ, LZ, NZ, EH, QL, TW, CM, BL, CL, YD, JZ, and XL: carried out data collections. WZ, LZ, AR, HS, and XZ: analyzed experimental results and wrote the manuscript.

### Conflict of Interest Statement

The authors declare that the research was conducted in the absence of any commercial or financial relationships that could be construed as a potential conflict of interest.
